# Low-Iron Diet-Induced Fatty Liver Development Is Microbiota Dependent and Exacerbated by Loss of the Mitochondrial Iron Importer Mitoferrin2

**DOI:** 10.3390/nu16121804

**Published:** 2024-06-08

**Authors:** Kendra A. Klag, Rickesha Bell, Xuan Jia, Alexandra Seguin, J. Alan Maschek, Mary Bronner, James E. Cox, June L. Round, Diane M. Ward

**Affiliations:** 1 Department of Pathology, Division of Microbiology and Immunology, University of Utah School of Medicine, Salt Lake City, UT 84132, USA; kendra.klag@path.utah.edu (K.A.K.); ricky.bell@path.utah.edu (R.B.); xuan.jia@biochem.utah.edu (X.J.); alexandra.seguin@path.utah.edu (A.S.); mary.bronner@path.utah.edu (M.B.); june.round@path.utah.edu (J.L.R.); 2 Metabolomics Core Research Facility, University of Utah, Salt Lake City, UT 84112, USA; alan.maschek@pharm.utah.edu (J.A.M.); jcox@cores.utah.edu (J.E.C.); 3 Huntsman Cancer Institute, Salt Lake City, UT 84112, USA; 4 Department of Biochemistry, University of Utah School of Medicine, Salt Lake City, UT 84132, USA

**Keywords:** iron, lipid, liver metabolism, microbiota, mitochondria

## Abstract

Iron deficiency is the number one nutritional problem worldwide. Iron uptake is regulated at the intestine and is highly influenced by the gut microbiome. Blood from the intestines drains directly into the liver, informing iron status and gut microbiota status. Changes in either iron or the microbiome are tightly correlated with the development of metabolic dysfunction-associated steatotic liver disease (MASLD). To investigate the underlying mechanisms of the development of MASLD that connect altered iron metabolism and gut microbiota, we compared specific pathogen free (SPF) or germ-free (GF) mice, fed a normal or low-iron diet. SPF mice on a low-iron diet showed reduced serum triglycerides and MASLD. In contrast, GF low-iron diet-fed mice showed increased serum triglycerides and did not develop hepatic steatosis. SPF mice showed significant changes in liver lipid metabolism and increased insulin resistance that was dependent upon the presence of the gut microbiota. We report that total body loss of mitochondrial iron importer Mitoferrin2 (*Mfrn2^−^/^−^*) exacerbated the development of MASLD on a low-iron diet with significant lipid metabolism alterations. Our study demonstrates a clear contribution of the gut microbiome, dietary iron, and Mfrn2 in the development of MASLD and metabolic syndrome.

## 1. Introduction

Iron is essential for almost all organisms as it is utilized for DNA replication and repair, oxygen transport, cellular respiration, proliferation, and differentiation [1,2,3]. In vertebrates, the gut is the major site of iron absorption and home to trillions of commensal microorganisms (microbiota) [4,5]. The microbiome of the gastrointestinal tract facilitates and battles for the acquisition of nutrients such as iron, influences host metabolism, and shapes host immunity [4,6,7,8,9]. Once iron is absorbed in the gut, it travels through the plasma through the portal vein to the liver. This major iron-sensing organ provides feedback signaling for either sufficient or insufficient iron in an organism. The hepatic portal vein also delivers gut-derived microbes and metabolites from the gut to the liver. Previous studies have shown that commensal gut microbes help maintain liver homeostasis but can also produce factors that contribute to liver damage under pathologic conditions [5,10,11]. The iron overload disorder hemochromatosis is highly correlated with liver diseases, including metabolic dysfunction-associated steatotic liver disease (MASLD), cirrhosis, liver cancer, as well as the development of metabolic syndrome, including type II diabetes mellitus (T2DM) [12,13,14,15]. In the setting of iron deficiency, we also see changes in metabolism and the gut microbiota, but this area of research has been under-explored. Studies suggest there is a “sweet spot” in iron homeostasis, and it is crucial to investigate how both dietary iron and microbiota composition impact metabolic health and disease of the liver and body [16,17,18,19,20,21].

Iron deficiency has historically been the number one nutritional problem worldwide, with approximately one to two billion affected individuals showing consequent anemia. Individuals with iron deficiency have changes in their gut microbiota compared to individuals with sufficient dietary iron [7,18,22]. Contrasting reports propose that lipid uptake may be increased or impaired in iron-deficiency anemia, suggesting that iron deficiency may influence the development of MASLD [20,23,24,25,26,27], which also affects a large percentage of the world population, with approximately one billion people manifesting signs of MASLD [20,28]. Excessive free fatty acid (FA) accumulation in MASLD is the result of increased FA availability to hepatocytes that exceeds its ability to remove FA by oxidation or export FA in lipoprotein particles (VLDL, LDL, and HDL). The origins of FA come from a combination of adipose tissue lipolysis, de novo FA synthesis, and diet [29]. Glucose and fatty acids derived from food are oxidized in the mitochondria via the TCA cycle and β-oxidation, respectively. Importantly, the TCA cycle and β-oxidation require iron as iron-sulfur (Fe-S), heme, or iron-containing enzymes. Since iron deficiency is the number one nutritional problem worldwide, and iron is essential for all organisms, we designed studies utilizing mouse models of iron deficiency (a low-iron diet) and altered mitochondrial homeostasis (Mitoferrin2 (Mfrn2) knockout) to better understand the interplay between nutritional iron, the gut microbiota, and mitochondrial iron homeostasis. Here, we show that specific pathogen free (SPF) mice on a low-iron diet developed MASLD with indications of metabolic syndrome, which significantly decreased in germ-free (GF) mice. SPF animals fed a low-iron diet had dramatic changes in liver lipid metabolism suggestive of altered mitochondrial FA β-oxidation, which requires sufficient mitochondrial iron. Iron import into the mitochondria is mediated predominantly by mitoferrins [30,31]. We show that loss of the mitochondrial iron importer Mitoferrin2 (Mfrn2) gave rise to MASLD even in SPF mice fed a normal-iron chow diet, supporting the idea that sufficient mitochondrial iron acquisition is needed to regulate fatty acid metabolism. When *Mfrn2^−/−^* SPF mice were placed on a low-iron diet, the MASLD was markedly exacerbated with decreased liver lipolysis and dramatic changes in liver lipid metabolism. Our results provide strong evidence of a tight correlation between iron homeostasis, liver metabolism, and mitochondrial metabolism that is influenced by the gut microbiota.

## 2. Materials and Methods

### 2.1. Animals

Mice: 6–8-week-old SPF, germ-free (GF) C57Bl/6 mice or SPF (WT), *Mfrn2^+/−^* and *Mfrn2^−^*^/*−*^ mice were fed standard rodent chow of 350 mg/kg iron or low-iron chow of 4–8 mg/kg (Harland, Teklad, Rockville, MD, USA) for 10 weeks. Animals were weighed then euthanized using isoflurane and cervical dislocation, as per IACUC animal protocols, and tissues were harvested. Body and liver weights were determined in all animals. The use of littermate controls allowed for proper control of the microbiota, as is standard in the field.

### 2.2. Histology

Tissues isolated from mice were fixed in formalin for a minimum of 24 h. A total of 5 µm sections were generated and stained with H&E by AML Laboratories (St. Augustine, FL, USA). Liver slides were blindly scored by a pathologist. Liver images were captured on an Olympus BX51 microscope (×10 objective) Fat depots were isolated from inguinal, subcutaneous, and brown fat (shoulder blades), fixed and processed similar to a liver histology.

### 2.3. Lipidomics

Extraction: All solutions are pre-chilled on ice. Tissues are transferred to labeled bead-mill tubes (1.4 mm, MoBio Cat# 13113-50, Qiagen, Chatsworth, CA, USA) where lipids are extracted in a solution of 225 µL MeOH containing internal standards (Avanti SPLASH LipidoMix, Merck, Darmstadt, Germany at 10 µL per sample) and 750 µL MTBE (methyl tert-butyl ether). The sample is homogenized in one 30 s cycle using the Omni Bead Ruptor followed by a rest on ice for 1 h. An addition of 188 µL PBS is made to induce phase separation. After centrifugation at 16,000× *g* for 5 min at 4 °C, the upper phases are collected and evaporated to dryness under a gentle nitrogen stream at room temperature. Lipid samples are reconstituted in 1 mL IPA/ACN/water (isopropyl alcohol, acetonitrile; 4/1/1, *v*/*v*/*v*) and transferred to an LC-MS vial with insert (Agilent 5182-0554 and 5183-2086, Santa Clara, CA, USA) for analysis. Concurrently, a process blank sample and pooled quality control (QC) sample is prepared by taking equal volumes (~50 µL) from each sample after final resuspension. The extraction protocol is based on Matyash et al. [32].

Mass Spectrometry Analysis of Samples: Lipid extracts are separated on a Waters Acquity UPLC CSH C18 1.7 µm 2.1 × 100 mm column maintained at 65 °C connected to an Agilent HiP 1290 Sampler, Agilent 1290 Infinity pump equipped with an Agilent 1290 Flex Cube, and Agilent 6530 Accurate Mass Q-TOF dual AJS-ESI mass spectrometer. For positive mode, the source gas temperature is set to 225 °C, with a gas flow of 11 L/min and a nebulizer pressure of 40 psig. VCap voltage is set at 3500 V, fragmentor at 110 V, skimmer at 85 V, and octopole RF peak at 750 V. For negative mode, the source gas temperature is set to 300 °C, with a drying gas flow of 11 L/min, and a nebulizer pressure of 30 psig. VCap voltage is set at 3500 V, fragmentor at 125 V, skimmer at 75 V, and octopole RF peak at 750 V. Samples are analyzed in a randomized order in both positive and negative ionization modes in separate experiments acquired with a scan range of *m*/*z* 100–1700. Mobile phase A consists of ACN:H_2_O (60:40 *v*/*v*) in 10 mM ammonium formate and 0.1% formic acid, and mobile phase B consists of IPA:ACN:H_2_O (90:9:1 *v*/*v*) in 10 mM ammonium formate and 0.1% formic acid. The chromatography gradient for both positive and negative modes starts at 15%; mobile phase B then increases to 30% B over 2.4 min; it then increases to 48% B from 2.4–3.0 min, then increases to 82% B from 3–13.2 min, then increases to 99% B from 13.2–13.8 min where it is held until 16.7 min and then returned to the initial condition and equilibrated for 5 min. Flow is 0.4 mL/min throughout, and injection volume is 1 µL for positive and 10 µL for negative modes. Tandem mass spectrometry is conducted using the same LC gradient at a collision energy of 25 V.

### 2.4. Analysis of Mass Spectrometry Data

QC samples (n = 8) and blanks (n = 4) are injected throughout the sample queue and ensure the reliability of acquired lipidomics data. Results from LC-MS experiments are collected using an Agilent Mass Hunter (MH) Workstation and analyzed using the software packages MH Qual B.07.00, MH Quant B.09.00, and Lipid Annotator B.01.00 (Agilent Technologies, Inc., Santa Clara, CA, USA). The data table exported from MHQuant is evaluated using Excel where initial lipid targets are parsed based on the following criteria: only lipids with relative standard deviations (RSD) less than 30% in the QC samples are used for data analysis. Additionally, only lipids with background AUC counts in process blanks that are less than 30% of QC are used for data analysis. The parsed Excel data is normalized to tissue mass. For volcano plots and heatmaps, the lipid abundances that were normalized to tissue mass were Pareto scaled and normalized.

### 2.5. LION/Web Lipid Ontology Analysis

The web-based tool LION (http://lipidontology.com/, accessed on 13 May 2024) was used in the ranking mode for lipidomic abundance data normalized to tissue mass [33,34].

### 2.6. Comments on Lipid Annotations

For a detailed description of lipid classification visit http://www.lipidmaps.org/data/classification/LM_classification_exp.php (accessed on 13 May 2024).

### 2.7. RNA Isolation and Real-Time Quantitative PCR (qPCR)

Tissue sections 0.5 cm in length were stored at −70 °C in 700 μL of RiboZol (VWR) or Trizol. RNA was isolated using the Direct-zol RNA MiniPrep Kit (Zymoresearch,, Irvine, CA, USA). cDNA was synthesized using a qScript cDNA synthesis kit (Quanta Biosciences, Beverly, MD, USA). qPCR was conducted using a LightCycler 480 SYBR Green I Master (Roche, Indianapolis, IN, USA). qPCR experiments were conducted on a Lightcycler LC480 instrument (Roche). Primers used in this study are listed in Table 1.

### 2.8. HOMA-IR, Serum Insulin, Blood Glucose

Mice were fasted for 6 h prior to being challenged with glucose. Fasting levels of glucose were detected using a Contour Glucose Meter (Bayer, Parsippany, NJ, USA) and Contour Glucose Strips (Bayer). One milligram of glucose per gram of body weight was injected intraperitoneally into animals at timepoint zero. Blood levels of glucose were measured at 5, 15, 30, 60, and 120 min time points using the glucose meter and strips.

### 2.9. Insulin ELISA

Serum was collected from 6 h-fasted mice, and insulin was measured using a mouse insulin ELISA kit (Crystal Chem, Grove Village, IL, USA). Serum samples were run in duplicate according to the manufacturer’s instructions.

### 2.10. Insulin Resistance Test

Mice were fasted for 6 h prior to being challenged with glucose. Fasting levels of glucose were detected using a Contour Glucose Meter (Bayer) and Contour Glucose Strips (Bayer). Insulin (0.75 U/kg of body weight) was injected intraperitoneally into animals at timepoint zero. Blood levels of glucose were measured at 5, 10, 15, 20, 25, 30, 40, and 60 min time points using the glucose meter and strips. Animals were removed from the experiment following a 150 μL i.p. injection of 25% glucose if blood glucose levels dropped to 30 mg/dL.

### 2.11. Triglyceride Analysis

Liver and serum triglycerides were assayed using detection kits ((Sigma, St. Louis MO, USA) TA101 and MAK043).

### 2.12. Statistical Analysis

All statistical analysis and graphing were done in GraphPad Prism 9.0 software. Outliers were identified in Prism using ROUT (Q = 1%). Data represent mean ± SD. Either one-way ANOVA or two-tailed unpaired student t tests were performed between groups as appropriate. Significance was noted at *p* ≤ 0.05 as *, *p* ≤ 0.01 as **, *p* ≤ 0.001 as ***, and *p* ≤ 0.0001 as ****.

## 3. Results

### 3.1. Decreased Nutritional Iron Availability Results in Increased Fat Deposition in the Liver

**Figure 1:**
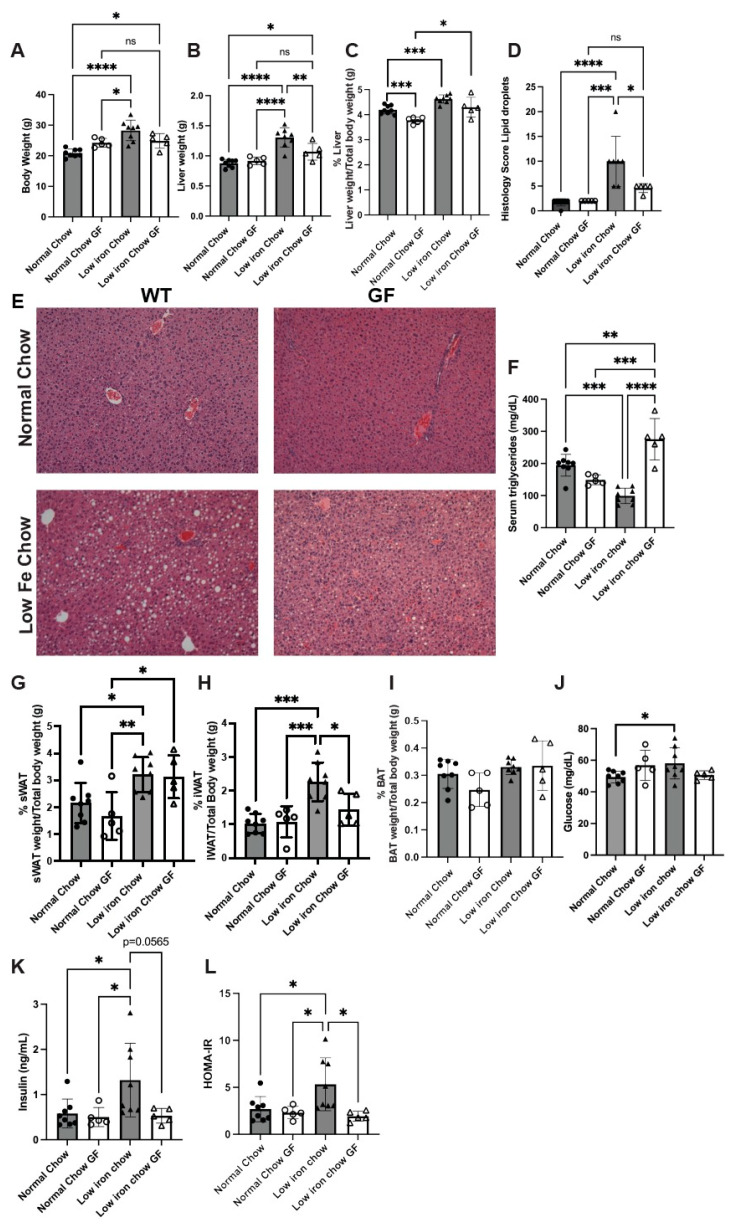
Low-iron diet causes increased MASLD that is dependent upon the presence of the gut microbiota. Six- to eight-week-old SPF (5–8) and GF female (5) mice were fed a normal (350 mg/kg chow) or a low-iron (4–8 mg/kg chow) diet for 10 weeks. Animals were euthanized according to IACUC approved protocols and (**A**) body weight, (**B**) liver weight, and (**C**) % liver of total body weight was determined. (**D**) Histologic scoring and (**E**) representative liver H&E staining of fatty livers from animals as in (**A**) are shown. (**F**) Serum TG for animals as in A were determined. (**G**) sWAT, (**H**) iWAT, and (**I**) BAT weights as a percent of total body weight were determined. (**J**) Glucose, (**K**) insulin, and (**L**) insulin resistance (HOMA-IR) were determined. N = 2 separate experiments with 5–8 animals/group/experiment. A representative experiment is shown. For all graphs, statistics were performed as described in Materials and Methods with *p* ≤ 0.05 considered significant. Significance was noted at *p* ≤ 0.05 as *, *p* ≤ 0.01 as **, *p* ≤ 0.001 as ***, and *p* ≤ 0.0001 as ****.

Iron homeostasis has long been appreciated for its effect on liver metabolism. There is a strong correlation between iron overload disorders and the development of non-alcoholic steatohepatitis/cirrhosis [35,36,37,38,39,40]. Indeed, this altered liver metabolism is also highly correlated with metabolic syndrome and the development of type 2 diabetes [13,36,37,41]. Recent studies suggest that the gut microbiome contributes to liver metabolic homeostasis [10,16,18]. Since both the microbiota and the host require iron, we wondered if limiting dietary iron would affect host liver metabolism and if the microbiota contributed to altered host metabolism. To address this question, we fed six-week-old SPF and germ-free (GF) C57Bl/6 female mice normal (350 mg/kg Fe) or low-iron (4–8 mg/kg Fe) chow for 10 weeks and measured liver and blood parameters of metabolism. We focused on female mice as previous studies have shown increased susceptibility to fatty liver disease in female mice [42]. Mice fed a low-iron diet showed increases in body weight, liver weight, and liver size as a percentage of total body weight compared to normal chow (Figure 1A–C). In contrast, GF animals fed a low-iron diet showed no change in body weight, although body weight was slightly elevated compared to SPF mice, with no change in liver weight but a slight increase in liver size as a percent of total body weight. Others have found that GF mice are resistant to weight gain on a high-fat diet, in part due to GF mice having poor absorption of lipids from the diet [29]. GF mice on a low-iron diet may also experience poor intestinal absorption of nutrients, which could contribute to a lack of body weight gain on a low-iron diet. We conclude that the increase in body weight, liver weight, and liver size relative to body weight due to the low-iron diet was dependent upon the presence of the gut microbiota. Liver histology revealed that the low-iron chow diet resulted in increased fat deposits in the livers of SPF animals, whereas the liver fat deposition was significantly decreased in GF animals fed a low-iron diet (Figure 1D,E). The absence of microbiota affecting lipid homeostasis was reflected in the serum triglyceride (TG) levels. SPF and GF mice fed a normal-iron diet showed no significant differences in serum TGs (Figure 1F). SPF mice showed reduced serum TGs when animals were nutritionally limited for iron. In contrast, GF mice showed dramatic increases in serum TGs, suggesting that the presence of the microbiota affects liver TG uptake from plasma. Others have also seen that GF mice on a hypercaloric diet do not gain weight compared to SPF mice, and that GF mice have elevated serum TGs [43].

Liver fat deposition can be a response to changes in fat metabolism at other locations. Therefore, we examined white fat (WAT) and brown fat depots (BAT) for changes in size. Visceral white fat depots were evaluated, including subcutaneous (sWAT) and inguinal (iWAT) depots. sWAT showed significant percentage increases relative to total body weight when animals were fed a low-iron diet, which also occurred in GF animals (Figure 1G). The iWAT depot sizes in animals fed the low-iron diet also increased in SPF animals but showed no significant difference due to diet in GF animals (Figure 1H). Brown fat (BAT), however, showed no significant changes due to diet or the presence of microbiota (Figure 1I). These results suggest that a low-iron diet also affects fat metabolism at other specific locations. Additional metabolic analyses revealed that SPF mice fed a low-iron diet had increased blood glucose levels (Figure 1J) and blood insulin levels (Figure 1K) and showed a corresponding increase in Homeostatic Model Assessment for Insulin (HOMA-IR) (Figure 1L), whereas GF mice did not show similar increases. Together, these results suggest that limited nutritional iron and gut microbiota contribute to the initiation of MASLD and the development of metabolic disease in mice.

### 3.2. Decreased Nutritional Iron and the Gut Microbiota Contribute to Altered Lipid Metabolism

**Figure 2:**
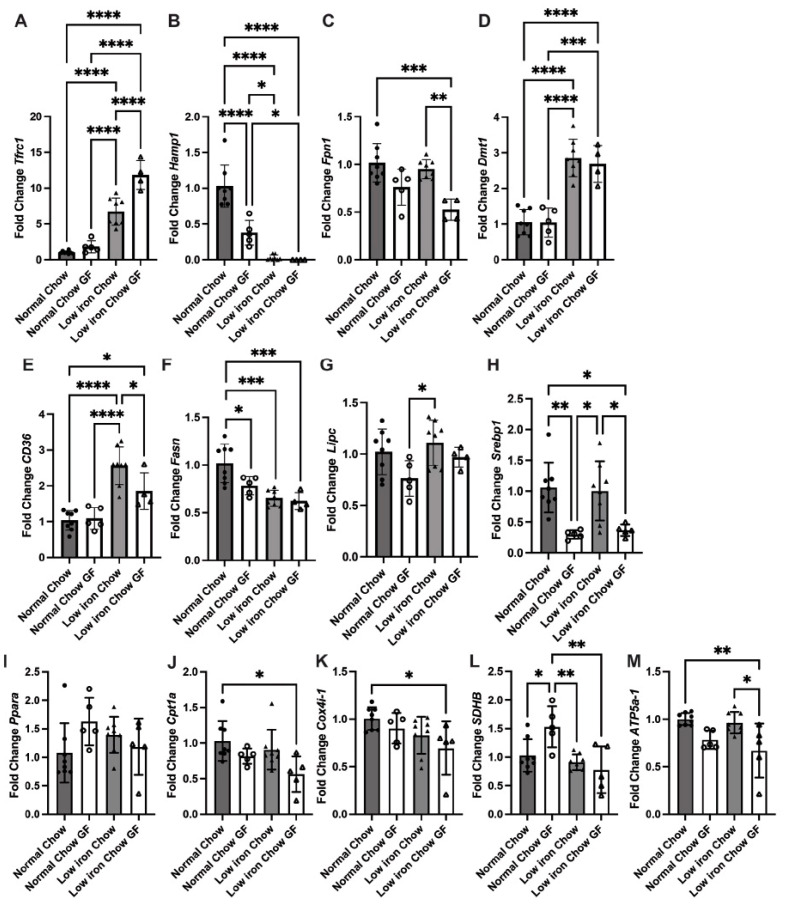
The low-iron diet limits iron availability and induces increased transcripts for long-chain fatty acid uptake in the liver. qPCR was performed on livers from animals as in Figure 1. qPCR of iron genes (**A**) *Tfrc1*, (**B**) *Hamp1,* (**C**) *Fpn1,* and (**D**) *Dmt1*. qPCR of (**E**) *CD36*, (**F**) *Fasn,* and (**G**) *Lipc*, (**H**) *Srebp-1c*, (**I**) *Pparα*, (**J**) *Cpt1a*, (**K**) *Cox4i-1*, (**L**) *SDHB,* and (**M**) *ATP5a1*. All transcripts are normalized to *B-Actin1* transcripts as described in Materials and Methods. N = 2 separate experiments with 5–8 animals/group/experiment. A representative experiment is shown. For all graphs, statistics were performed as described in Materials and Methods with *p* < 0.05 considered significant. Significance was noted at *p* ≤ 0.05 as *, *p* ≤ 0.01 as **, *p* ≤ 0.001 as ***, and *p* ≤ 0.0001 as ****.

To confirm that the low-iron diet altered iron homeostasis in the liver, we measured transcripts for some iron responsive genes, including transferrin receptor 1 (*Tfrc1*), iron regulatory peptide hepcidin (*Hamp*), iron exporter ferroportin1 (*Fpn1*), and divalent metal transporter 1 (*Dmt1*). *Hamp1* encodes for the anti-microbial peptide hepcidin as well as having activity in iron homeostasis [44,45,46,47,48,49]. As expected, animals fed a low-iron diet showed increased expression of liver *Tfrc1* and *Dmt1* for iron acquisition and decreased expression of *Hamp1,* which controls iron export into plasma from tissues, whereas *Fpn1* expression was unchanged by the low-iron diet (Figure 2A–D). The absence of microbiota resulted in increased *Tfrc1* transcripts and a trend toward decreased expression of *Fpn1* compared to SPF animals. The mechanisms responsible for transcriptional changes in the liver mediated by the microbiota have yet to be fully elucidated, but these changes suggest that the livers of GF mice have less bioavailable iron. As expected, the low-iron diet and the absence of a microbiota were additive in reducing expression of *Hamp1.* The transcriptional responses confirm that the low-iron diet resulted in significant changes in liver iron and revealed novel transcriptional changes in iron responsive genes *Tfrc1* and *Fpn* that were specific to the absence of the microbiota. These microbiota-specific changes highlight the ability of the GF mouse to sense/utilize/store iron in a different way, which may suggest a mechanism for protection from fatty liver development on a low-iron diet.

To identify mechanistically how lipid accumulation in the liver is altered in response to a low-iron diet, we measured liver transcripts for long-chain FA uptake (*CD36*) [50], de novo FA biosynthesis (*Fasn*) [51,52], and hepatic lipase (*Lipc*), which helps keep fat-transporting molecules in balance by regulating the formation of low-density lipoproteins (LDLs) and the transport of high-density lipoproteins (HDLs) [53]. *CD36* transcripts were increased in response to the low-iron diet only in the SPF mice but were not significantly increased in the GF animals (Figure 2E). *Fasn* transcripts were reduced in GF mice on normal chow and decreased in both SPF and GF mice in response to the low-iron diet (Figure 2F). *Lipc* transcripts were not significantly changed in animals fed a low-iron diet (Figure 2G). These results suggest that when nutritional iron is limiting, the liver responds by increasing long-chain FA uptake through CD36 in a manner exacerbated by the microbiome’s presence. The reduction in *Fasn* transcripts in GF compared to SPF mice fed normal chow may provide a mechanism for some protection from MASLD in the nutritionally iron-limited mice. Indeed, previous studies have demonstrated that GF mice show reduced lipid absorption [29] and a resistance to diet-induced obesity through increased FA metabolism [54].

### 3.3. GF Animals Express Markedly Reduced Levels of Lipogenesis Genes SREBP-1c and Fasn

FA metabolism can be controlled by two classes of transcriptional regulators: peroxisome proliferator-activated receptors (PPARs) [55,56] and sterol regulatory element binding proteins (SREBPs) [57,58,59]. In hepatocytes, PPARα is responsible for lipolytic enzyme gene expression (e.g., *Lipc*), and SREBP-1c regulates lipogenesis (e.g., *Fasn*). That *Fasn* expression was decreased in GF mice on normal chow suggests that the GF consequences of liver lipid metabolism may be regulated by lipogenesis through SREBP-1c. Indeed, GF animals showed a dramatic reduction in *Srebp-1c* transcripts even on normal chow (Figure 2H). *Pparα* transcripts were elevated in GF mice but were not significantly altered by dietary iron (Figure 2I). The fact that SPF animals showed increased liver lipid droplets on a low-iron diet and increased WAT depot sizes (Figure 1G–I) supports the idea that there is reduced lipolysis in several tissues in response to a low-iron diet. While Figure 2 only assesses gene transcription and not functional changes in protein levels, the cumulative transcriptional changes in many genes related to lipid metabolism like *CD36* and *Srebp1* and the liver histological changes seen in Figure 1D,E suggest that these changes in transcription do have a functional consequence. Furthermore, loss of CD36 has been shown to decrease mRNA levels of *Srebp1* [52], and here we report that both *CD36* and *Srepb1* transcripts are downregulated in GF mice on a low-iron diet compared to SPF. Together, these observations support that dietary iron levels contribute to the development of MASLD and the metabolic syndrome, which is prevented in GF mice.

FA oxidation occurs in the mitochondria. Under conditions where TGs accumulate in hepatocytes, FA oxidation may likely be insufficient due to import defects or reduced FA oxidation. Increased FA oxidation is also known to give rise to oxidative stress, especially when mitochondrial function is compromised [60]. Thus, reduced FA oxidation might protect against cellular damage during the development or advancement of MASLD. To determine how FA oxidation might be changed, we examined the expression of mitochondrial carnitine palmitoyltransferase 1 (*Cpt1a*) (the mitochondrial long-chain FA oxidation enzyme) [61], Cytochrome C oxidase (*Cox4i-1*) (oxidative phosphorylation complex IV), Succinate dehydrogenase B (*SDHB*) (oxidative phosphorylation complex II), and a subunit of mitochondrial ATP synthase (*ATP5a-1*) (complex V) [62]. *Cpt1a* and *Cox4i-1* transcripts were altered only in GF animals fed a low-iron diet (Figure 2J,K), which is predictive of reduced FA entry into the mitochondrial matrix and may lead to less FA oxidation. *SDHB* transcripts (Complex II) were increased in GF mice fed normal chow, but again responded by decreasing in GF animals fed a low-iron diet (Figure 2L). *ATP5a-1* transcripts encoding for complex V were slightly lower in GF mice but unaffected by the low-iron diet (Figure 2M).

### 3.4. Liver Lipid Metabolism Is Altered by Both the Microbiota and Dietary Iron Limitation

**Figure 3:**
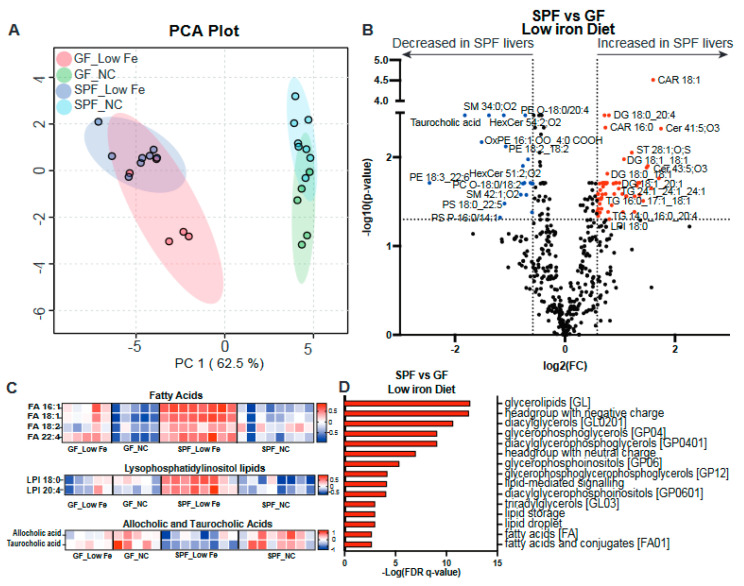
SPF mice fed a low-iron diet show increased levels of fatty acids and LPI. Lipidomic analysis was performed on the livers of SPF and GF mice fed normal or low-iron chow. (**A**) Principle Component Analysis of the liver lipidomics. NA = Normal Chow. (**B**) Volcano plot analysis of changes in liver lipids in SPF and GF mice fed a low-iron diet. (**C**) Heatmaps of FA, LPI, and allocholic and taurocholic acids in SPF and GF mice on normal or low-iron chow. (**D**) LION analysis of SPF and GF changes in response to the low-iron diet.

To better understand the total changes in lipids in the liver in response to nutritional iron limitation and/or the absence of a microbiota, we performed lipidomic analysis using mass spectrometry on livers from SPF and GF mice fed a normal or low-iron diet. Principal Component Analysis (PCA) revealed significant changes in the liver lipidome due to alterations in the nutritional iron diet and, to a lesser extent, the absence of a gut microbiota (Figure 3A, Supplementary Table S1). Comparison of the SPF vs GF liver lipidomes fed a low-iron diet revealed significant increases in FA, diacylglycerides (DG), TG, and some ceramides (Figure 3B,C). Indeed, heatmap analysis identified that while SPF mice fed a low-iron diet showed dramatic FA increases, GF mice’s FA increases were much less pronounced (Figure 3C). In addition, SPF mice on a low-iron diet also showed increased levels of lysophosphatidylinositols (LPI), while GF mice did not (Figure 3C). That GF mice on a low-iron diet did not show increases in LPI suggests that the microbiome contributes to regulating the levels of this bioactive lipid, which has been shown to play a role in metabolic disorders [63]. In addition, both bile acids, allocholic and taurocholic acids, (TCA) were decreased by the low-iron diet, and more so in the SPF than the GF mice (Figure 3C). Interestingly, TCA administration has been shown to protect from hepatic lipid accumulation [64]. Lipid Ontology (LION) enrichment analysis revealed many significant differences in lipid classes, biophysical properties, lipid functions, and organelle associations between SPF and GF mice on a low-iron diet, indicating that the microbiome substantially impacts liver lipid metabolism (Figure 3D, Supplementary Table S2).

### 3.5. Loss of Mitochondrial Iron Importer Mitoferrin2 Results in Increased MASLD That Is Exacerbated by a Low-Iron Diet

**Figure 4:**
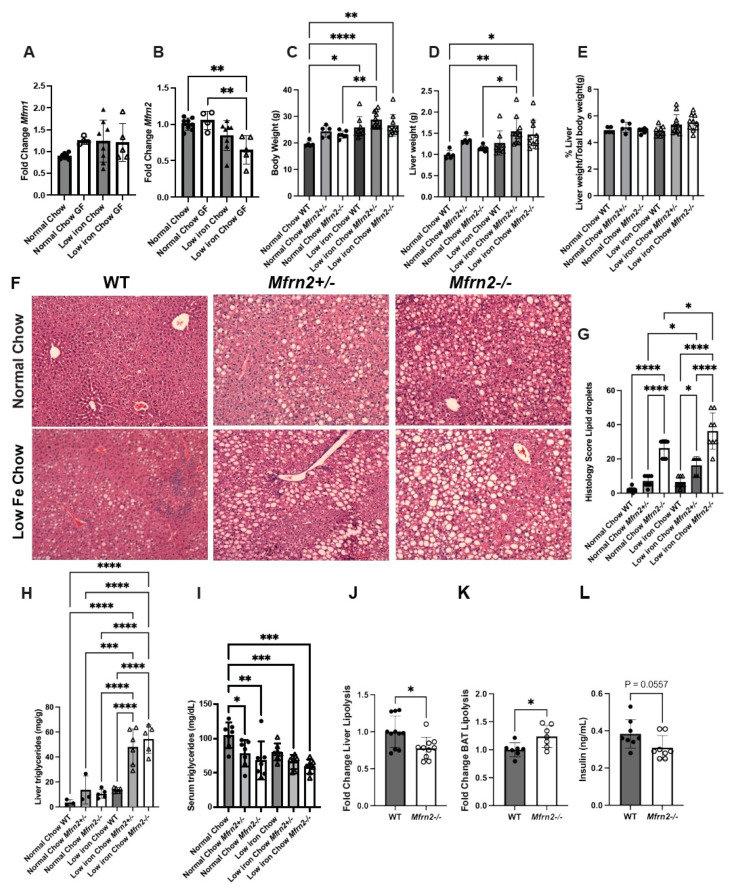
Loss of mitochondrial iron importer Mfrn2 results in MASLD. qPCR on (**A**) *Mfrn1*, (**B**) *Mfrn2,* and *b-Actin* was performed on animals as in Figure 1. Transcripts were normalized relative to *b-Actin*. Six-to-eight-week-old female *Mfrn2*^+/+^, *Mfrn2*^+/−^, and *Mfrn2*^−/−^ mice were maintained on a normal chow (350 mg/kg iron) or a low-iron chow (4–8 mg/kg iron) for 10 weeks (n = 3–10 mice/genotype/diet), and (**C**) body weight, (**D**) liver weight, and (**E**) liver/body weight ratios were determined as a percent of total body weight. (**F**) H&E staining on livers from animals as in (**C**). (**G**) Histologic quantification of liver lipid accumulation. Representative images are shown for each genotype on each diet. (**H**) Liver and (**I**) serum triglycerides were measured as described in Materials and Methods. (**J**) Liver lipolysis, (**K**) BAT lipolysis, and (**L**) serum insulin levels were determined in WT and *Mfrn2*^−/−^ mice. A representative experiment is shown. N = 2–3 separate experiments. For all graphs, statistics were performed as described in Materials and Methods with *p* ≤ 0.05 considered significant. Significance was noted at *p* ≤ 0.05 as *, *p* ≤ 0.01 as **, *p* ≤ 0.001 as ***, and *p* ≤ 0.0001 as ****.

Much of lipid metabolism is regulated by the mitochondria, and many mitochondrial functions depend upon the presence of iron in the mitochondria for the production of heme and Fe-S clusters, prosthetic groups that populate many enzymes required for the electron transport chain (ETC), mitochondrial homeostasis, and β-oxidation. Mitochondrial iron import is mediated predominantly by Mitoferrins (Mfrns) [30,65,66,67]. Previously, we and others determined that Mfrn1 was necessary for erythroid development and that its loss dramatically affected mitochondrial iron levels in those hematopoietic tissues [31,68]. We further showed that Mfrn1 and Mfrn2 are important for cell proliferation [30]. Since FAs are oxidized in the mitochondria, we postulated that the levels of Mfrns might play a role in the development of MASLD. Expression analyses of *Mfrns* showed that *Mfrn1* transcripts were unaffected by the gut microbiota’s presence or absence or the low-iron diet (Figure 4A). In contrast, *Mfrn2* expression was dramatically reduced in GF mice fed a low-iron diet (Figure 4B).

Based upon the changes in *Mfrn2* transcripts measured and the fact that we noticed increased lipid droplets in the absence of Mfrn2 in our previous study [30], we examined metabolic changes in WT (SPF) and *Mfrn2*^−^*^/^*^−^ mice fed normal or low-iron chow. Loss of Mfrn2 (heterozygous and homozygous) resulted in increased body weight that was exacerbated by the low-iron diet (Figure 4C). Liver size was not significantly increased by loss of Mfrn2, but the low-iron diet resulted in a trend toward increased liver size (Figure 4D). Liver/body weight ratios were unaffected by the loss of *Mfrn2* (Figure 4E). *Mfrn2^−/−^* animals showed increased hepatocyte lipid droplets on normal-iron chow, which dramatically increased when animals were fed low-iron chow (Figure 4F,G). This was most dramatic in female mice, but male mice also showed increased fatty liver histology (Supplementary Figure S1). Increased fatty livers were seen even in *Mfrn2^+/−^* animals, suggesting haploinsufficiency. Altered lipid homeostasis was seen in whole body *Mfrn2* knockout animals but was not observed in tissue-specific (hepatocyte (*Alb-Cre*) or adipocyte (*ADP-Cre*)) *Mfrn2f/f* (knockout) mice (Supplementary Figure S2). In agreement with the lipid droplet accumulation, liver TGs were elevated in *Mfrn2^−/−^* mice and were dramatically increased when animals were fed a low-iron diet (Figure 4H). Serum TGs were reduced in normal-chow-fed animals due to the loss of Mfrn2, but the levels were not significantly decreased in *Mfrn2^+/−^* nor *Mfrn2^−/−^* mice fed a low-iron diet (Figure 4I). Corresponding to increased liver TG accumulation, we observed decreased liver lipolysis and increased BAT lipolysis in *Mfrn2^−/−^* mice compared to WT controls (Figure 4J,K), suggesting that loss of Mfrn2 alters lipid metabolism differently in these tissues. We also noted that serum insulin levels were slightly reduced in the absence of Mfrn2 (Figure 4L), while HOMA-IR and serum glucose were unaffected (Supplementary Figure S3).

**Figure 5:**
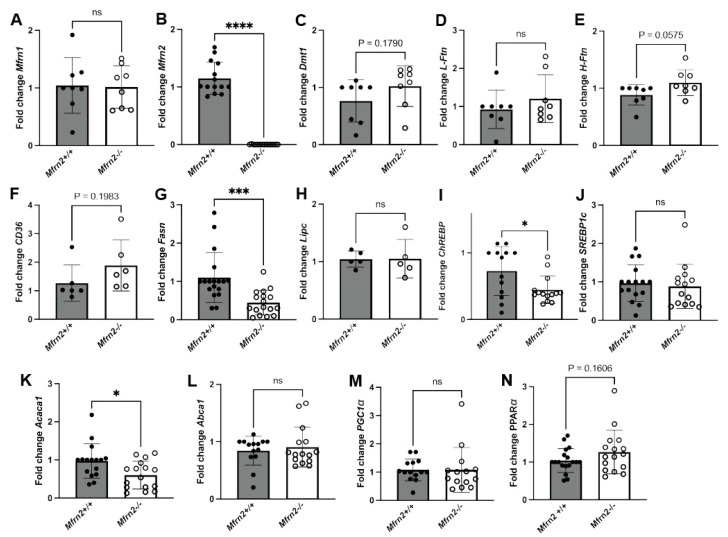
Loss of Mfrn2 alters expression of FA synthesis and lipogenic pathways. Liver mRNA was isolated from three-month-old female *Mfrn2*^+/+^, *Mfrn2*^+/−^, and *Mfrn2*^−/−^ mice maintained on a low-iron diet for 10 weeks and qPCR performed for (**A**) *Mfrn1*, (**B**) *Mfrn2*, (**C**) *Dmt1*, (**D**) *L-Ftn*, and (**E**) *H-Ftn*. qPCR was performed for lipogenesis genes (**F**) *CD36*, (**G**) *Fasn,* and (**H**) *Lipc;* energy metabolism regulators (**I**) *ChREBP* and (**J**) *SREBP1c*; lipid synthesis genes (**K**) *Acaca1* and (**L**) *Abca1;* and master lipid regulators (**M**) *PGC1α* and (**N**) *PPARα* using primers described in Table 1. N = 5–15. For all graphs, statistics were performed as described in Materials and Methods with *p* ≤ 0.05 considered significant. Significance was noted at *p* ≤ 0.05 as *, *p* ≤ 0.001 as ***, and *p* ≤ 0.0001 as ****.

We next examined gene expression in the liver of WT and *Mfrn2^−/−^* mice fed a low-iron diet. There was no difference in *Mfrn1* expression in the liver, while *Mfrn2* transcripts were absent as expected (Figure 5A,B). There were also minimal differences in iron-related gene expression for *Dmt1*, light chain ferritin (*L-Ftn*), or heavy chain ferritin (*H-Ftn*) (cytosolic iron storage proteins) in *Mfrn2^−/−^* mice compared to WT controls fed a low-iron diet (Figure 5C–E). We showed that the low-iron diet induced increased expression of *CD36* in SPF and GF mice (Figure 2E). Loss of Mfrn2 trended toward increased *CD36* transcripts over WT controls in low-iron diet fed animals (Figure 5F). In contrast, de novo lipogenesis gene *Fasn* expression was significantly reduced in low-iron diet fed *Mfrn2^−/−^* mice compared to WT controls (Figure 5G). Loss of Mfrn2 did not affect the expression of *Lipc* (Figure 5H). To guide in determining the mechanism of increased lipid droplets in *Mfrn2^−/−^* mice, we measured transcripts for master transcriptional regulators of lipogenesis *ChREBP* and *SREBP1c*. There was a reduction in *ChREBP* transcripts but no difference in *SREBP-1c* transcripts in the livers of *Mfrn2^−/−^* mice fed a low-iron diet (Figure 5I,J). However, the SREBP-1c target gene Acetyl-CaA carboxylase alpha (*Acaca1)*, which acts as a rate-limiting enzyme in the de novo synthesis of FA, showed reduced transcripts in *Mfrn2^−/−^* livers (Figure 5K). Transcripts for the cholesterol efflux regulatory protein *Abca1* were unaltered (Figure 5L). No changes were observed in liver transcript levels of the master regulator of mitochondrial biogenesis *Pgc1-a,* and there was only a trend toward increased expression of *Pparα* in the absence of Mfrn2 (Figure 5M,N). That the lipogenic genes transcripts are not increased in the liver supports that there is not an increased synthesis in the absence of Mfrn2 or a low-iron diet, but rather there is a change in hepatocyte FA oxidation that gives rise to increased steatosis.

### 3.6. Low-Iron Diet and Loss of Mfrn2 Are Additive for Altered Liver Lipid Homeostasis

**Figure 6:**
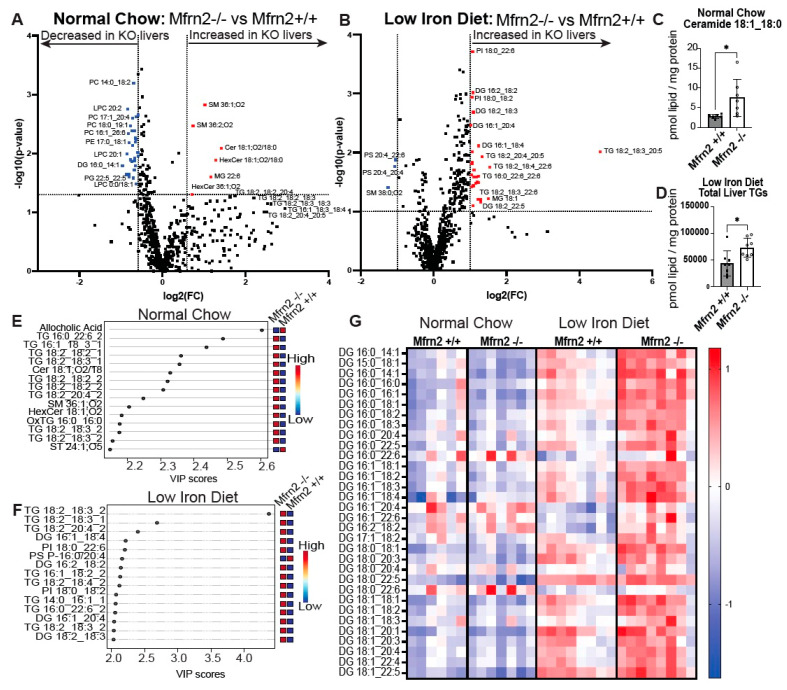
*Mfrn2^−/−^* mice show elevated TG, DG, and ceramides on a low-iron diet. Lipidomic analysis was performed on the livers of WT (SPF) and *Mfrn2^−/−^* mice fed normal or low-iron chow. Volcano plot analysis of changes in liver lipids in *Mfrn2^−/−^* and WT mice fed a (**A**) normal chow diet or (**B**) a low-iron diet. (**C**) Liver ceramide 18:1_18:0 levels in WT and *Mfrn2^−/−^* mice fed a normal chow diet. (**D**) Total liver TG levels in WT and *Mfrn2^−/−^* mice fed a low-iron diet. PLS VIP scores for lipid species in *Mfrn2^−/−^* and WT mice fed a (**E**) normal chow diet or (**F**) a low-iron diet. (**G**) Heatmaps of diacylglycerol (DG) changes. Significance was noted at *p* ≤ 0.05 as *.

MASLD is characterized by the accumulation of different species of lipids, some of which are protective, such as TG, and some that are more toxic, such as diacylglycerol (DG), acylcarnitines, and ceramides, which drive inflammation, hepatocyte cell death, and fibrosis [69,70]. Contrasting reports suggest that FA uptake, lipogenesis, or FA oxidation are increased or impaired in iron-deficiency anemia, underscoring how iron changes can affect multiple lipid homeostasis pathways [71,72,73]. It is also suggested that iron deficiency anemia or iron overload alters the transcriptional profile of genes that encode proteins involved in lipid homeostasis. To identify potential pathways/molecules that may be involved in early MASLD development in *Mfrn2^−/−^* mice, we performed lipidomics on animals fed normal or low-iron chow for 10 weeks, similar to Figure 3. *Mfrn2^−/−^* mice fed normal chow showed increased levels of TGs and ceramides compared to WT animals (Figure 6A,C,E, Supplementary Table S3). While TG increased in WT animals fed a low-iron diet, *Mfrn2^−/−^* mice fed a low-iron diet continued to show higher levels of TG as well as increased levels of the toxic lipid DG (Figure 6B,D,F,G). Importantly, these lipids are found to be elevated in patients with MASLD [74,75,76]. These results lead to the conclusion that Mfrn2 is necessary to control fat metabolism even when Mfrn1 is present. These results also demonstrate that the loss of mitochondrial iron import through Mfrn2 alters lipid metabolism even before dietary iron limitation. Together with the gene expression analyses, our results suggest that changes in iron homeostasis, either due to dietary limitations or mitochondrial iron import, affect liver FA oxidation and may be part of the multiple hit hypothesis for contributing to the development and progression of MASLD and metabolic disease.

## 4. Discussion

The gut microbiota contributes to organismal iron homeostasis and liver metabolism. Limiting iron in the diet affects the composition of the gut microbiota, intestinal iron absorption and tissue-specific iron depots [4,5,6,7,8,18,77,78,79]. Both the amount of iron in the diet and the ability of the host to absorb iron directly influence the composition of the microbiota. Here, we report that dietary iron limitation causes increased liver TG accumulation, the development of MASLD, and metabolic syndrome in mice. We found that the development of liver and metabolic disease is dependent on the presence of the microbiota, as GF mice are protected from low-iron diet-induced MASLD. Previous studies have also shown that GF mice are protected from hypercaloric-induced liver steatosis [43]. We provide evidence that low dietary iron dramatically impacts the liver lipidome, leading to the accumulation of toxic lipids such as DG, ceramides, lysophosphatidylinositol lipids, and diminished protective lipids such as TCAs. The absence of a gut microbiota reduced the accumulation of these liver FA species seen in SPF mice fed a low-iron diet, suggesting that certain microbes may negatively impact liver metabolism in the setting of a low-iron diet. We determined that GF mice had a robust reduction in the expression of the master regulator of lipogenesis *SREBP-1c* [80], and the downstream target of FA synthesis, *Fasn*. Additively, on a low-iron diet, the liver of GF mice had a reduced capacity for lipid absorption, as evidenced by decreases in the FA transporter CD36. Together, these results provide a mechanism to explain reduced liver TG accumulation in GF mice. Our observations also suggest that alterations in organismal iron homeostasis either through dietary iron acquisition, organ/tissue iron storage, and/or microbiome constituents provide an iron-dependent multi-hit that influences the development of MASLD and metabolic syndromes much like the predicted multi-hit hypothesis [12,60,81,82]. Of note, the low-iron diet used in these studies was given for ten weeks to young animals; more long-term use of a low-iron diet in aged animals may better reflect what happens in humans that are chronically limited for dietary iron and be a model for Metabolic Dysfunction-Associated Steatohepatitis (MASH). Furthermore, it may prove valuable to combine the low-iron diet with other dietary metabolic challenges, such as high fats and or sugars, both hallmarks of the Western diet and metabolic disease. Our results support that there is a “sweet spot” in iron homeostasis where lipid metabolism is “normal,” but deviations in iron homeostasis can drive the development of metabolic disorders.

The current study guides potential therapeutic approaches to altered liver lipid metabolism and the development of MALSD. First, changing the gut microbiome can strongly influence the development of metabolic syndromes. This poses the question of whether metabolically protective or detrimental microbes that contribute to an individual’s susceptibility to disease can be identified. Subsequently, these microbes could be supplemented or eradicated as a therapeutic avenue. Second, several SREBP inhibitors are available that might mimic the lipid metabolism consequences of an altered gut microbiome [83]. Caution must be taken with completely inhibiting SREBP as previous studies have shown that loss of the SREBP pathway can be detrimental in MASLD and steatohepatitis, resulting in increased liver injury [84]. Thus, SREBP inhibitors must be carefully titrated to allow some function in liver lipid homeostasis. An area for future investigation in this project should explore how the microbiota and a low-iron diet modulate levels of oxidative stress. Oxidative stress has been shown to be exacerbated by iron deficiency anemia [85]. Additionally, microbial metabolites can promote or protect from MASLD through the regulation of oxidative stress [86].

We discovered that the loss of the microbiome (GF) together with a low-iron diet resulted in decreased expression of the mitochondrial iron importer Mfrn2. This was surprising because iron import into the mitochondria is essential for the formation of Fe-S clusters, heme synthesis, and mitochondrial oxidation [3,67]. Reduced *Mfrn2* expression in GF mice suggests that under limited dietary iron GF liver mitochondrial metabolism is already altered, and bringing in iron through Mfrn2 might be detrimental for hepatocytes. We previously showed that the loss of Mfrn2 did not affect liver mitochondrial iron levels under a normal iron diet but did affect mitochondrial iron levels when animals were placed on a low-iron diet, with consequent reductions in oxidative phosphorylation complex proteins that require iron [30]. In that same study, we determined that Mfrn1 and Mfrn2 were important for liver regeneration after partial hepatectomy. In the current study, we uncover the importance of Mfrn2 in lipid metabolism. Total body *Mfrn2^−/−^* female mice showed elevated liver TG and altered liver lipid metabolism upon exposure to a low-iron diet as determined by lipidomics, with a corresponding development of MASLD. The fact that mice heterozygous for *Mfrn2* deletion showed lipid metabolism defects further demonstrates that Mfrn2 is vital in lipid homeostasis and that other metal transporters cannot compensate for the loss of Mfrn2 under iron-limited conditions. It is important to emphasize that this current study shows that mitochondrial iron import through *Mfrn1* is insufficient to maintain lipid homeostasis. Lipid homeostasis is regulated by several tissues, including liver, fat, muscle, and brain. It may be that several tissues contribute to the lipidomic changes seen due to the loss of Mfrn2, and further studies are merited to better understand the mechanistic defects.

Studies have implicated iron in hepatic steatosis, although most studies have correlated iron overload with increased MASLD [12,87,88]. Our results demonstrate that dietary iron limitation can be causal to the development of hepatic steatosis as WT (SPF) animals showed increased liver TG when placed on a low-iron diet. The accumulation of TG may act as a protective mechanism to store energy when iron is limiting but may also lead to deleterious lipid metabolism changes over time. What needs further investigation is what products or individuals from the gut microbiome contribute to changes in liver lipid metabolism and how dietary iron alters those parameters [89].

## 5. Conclusions

In conclusion, this study identified unique components involved in the multifactorial development of MASLD and metabolic syndrome. Our data highlight how the most common nutritional deficit in the world, iron deficiency, may be a driver of MASLD. We show that alterations in the gut microbiome can protect from the development of MASLD caused by low dietary iron. Additionally, the role of limited iron in liver lipid metabolism is deepened by our findings that the mitochondrial iron importer Mfrn2 plays a crucial role in lipid metabolism and that *Mfrn2^−/−^* mice provide a novel model to study MASLD over time without altering diet.

## Figures and Tables

**Table 1:** Primers for qPCR.

Gene		Primer Sequence	Reference
*Abca1*	Forward	AAAACCGCAGACATCCTTCAG	Origene
	Reverse	CATACCGAAACTCGTTCACCC	*β*
*Acaca1*	Forward	TGTACAAGCAGTGTGGGCTGGCT	*β*
	Reverse	CCACATGGCCTGGCTTGGAGGG	*β*
*ATP5a1*	Forward	TGGTGAAGAGACTGACGGATGC	*β*
	Reverse	TCAAAGCGTGCTTGCCGTTGTC	*β*
*β Actin*	Forward	GACGGCCAAGTCATCACTATTG	*β*
	Reverse	CCACAGGATTCCATACCCAAGA	*β*
*CD36*	Forward	GGACATTGAGATTCTTTTCCTCTG	*β*
	Reverse	GCAAAGGCATTGGCTGGAAGAAC	*β*
*ChREBP*	Forward	AGATGGAGAACCGACGTATCA	*β*
	Reverse	ACTGAGCGTGCTGACAAGTC	*β*
*Cox4i1*	Forward	TCATTGGCTTCACTGCGCTCGT	*β*
	Reverse	TCCAGCATTCGCTTGGTCTGCA	*β*
*CPT1a*	Forward	GGCATAAACGCAGAGCATTCCTG	*β*
	Reverse	CAGTGTCCATCCTCTGAGTAGC	*β*
*Dmt1*	Forward	AGCTAGGGCATGTGGCACTCT	*β*
	Reverse	ATGTTGCCACCGCTGGTATC	*β*
*Fasn*	Forward	GCTGCGGAAACTTCAGGAAAT	*β*
	Reverse	AGAGACGTGTCACTCCTGGACTT	*β*
*Fpn*	Forward	CCATAGTCTCTGTCAGCCTGCT	*β*
	Reverse	CTTGCAGCAACTGTGTCACCGT	*β*
*L-Ftn*	Forward	ATGACCTCTCAGATTCGTCAG	*β*
	Reverse	ATTCGCGGAAGAAGTGGCCTA	*β*
*H-Ftn*	Forward	CTCCTACGTCTATCTGTCTATG	*β*
	Reverse	ATTCGGCCACCTCGCTGGTTCT	*β*
*Hamp*	Forward	CAGCACCACCTATCTCCATCAAC	*β*
	Reverse	CAGATGGGGAAGTTGGTGTCTC	*β*
*Lipc*	Forward	CTTCCAGCCTGGCTGCCACTT	*β*
	Reverse	GCAAGGAGTCAATGAAGAGGTGC	*β*
*Mfrn1*	Forward	TTGAATCCAGATCCCAAAGC	*β*
	Reverse	GTTTCCTTGGTGGCTGAAAA	*β*
*Mfrn2*	Forward	TCGTCAAGCAGAGGATGCAGAT	*β*
	Reverse	GTTAAAGTGCTCTTGCAGGAAC	*β*
*PGC1* *α*	Forward	GAATCAAGCCACTACAGACACCG	*β*
	Reverse	CATCCCTCTTGAGCCTTTCGTG	*β*
*Pparα*	Forward	ACCACTACGGAGTTCACGCATG	*β*
	Reverse	GAATCTTGCAGCTCCGATCACAC	*β*
*SDHB*	Forward	TGCGGACCTATGGTGTTGGATG	*β*
	Reverse	CCAGAGTATTGCCTCCGTTGATG	*β*
*Srebp-1c*	Forward	CGACTACATCCGCTTCTTGCAG	*β*
	Reverse	CCTCCATAGACACATCTGTGCC	*β*

Primers used for qPCR assays.

## Data Availability

The original contributions presented in the study are included in the article/Supplementary Material, further inquiries can be directed to the corresponding authors.

## Supplementary Materials

The following supporting information can be downloaded at: https://www.mdpi.com/article/10.3390/nu16121804/s1, Figure S1: The low iron diet reveals *Mfrn2^−/−^* male mice also show MASLD; Figure S2: Tissue specific loss of *Mfrn2* in hepatocytes or adipocytes does not result in MASLD; Figure S3: Loss of *Mfrn2* does not affect serum glucose levels nor HOMA-IR; Table S1: GF vs. SPF Normal Chow vs. Low Iron Chow lipidomics; Table S2: LION SPF vs. GF Low Iron Chow lipidomics; Table S3: *Mfrn2^−/−^* vs. *Mfrn2^+/+^* Normal Chow vs. Low Iron Chow lipidomics.
