# Supplementary material for: Low-Iron Diet-Induced Fatty Liver Development Is Microbiota Dependent and Exacerbated by Loss of the Mitochondrial Iron Importer Mitoferrin2
Source: Nutrients. 2024 Jun 8;16(12):1804. doi: 10.3390/nu16121804 (PMC11206261; doi:10.3390/nu16121804)
Supplement: Supplementary file 1 [file nutrients-16-01804-s001.zip › Supplemental Material.pdf]

Supplemental Tables

Supplemental Table S1 GF vs SPF Normal Chow vs Low Iron Chow lipidomics

Supplemental Table S2 LION SPF vs GF Low Iron Chow lipidomics

Supplemental Table S3 Mfrn2-/- vs Mfrn2+/+ Normal Chow vs Low Iron Chow lipidomics

Supplemental Figures

Sup Figure 1

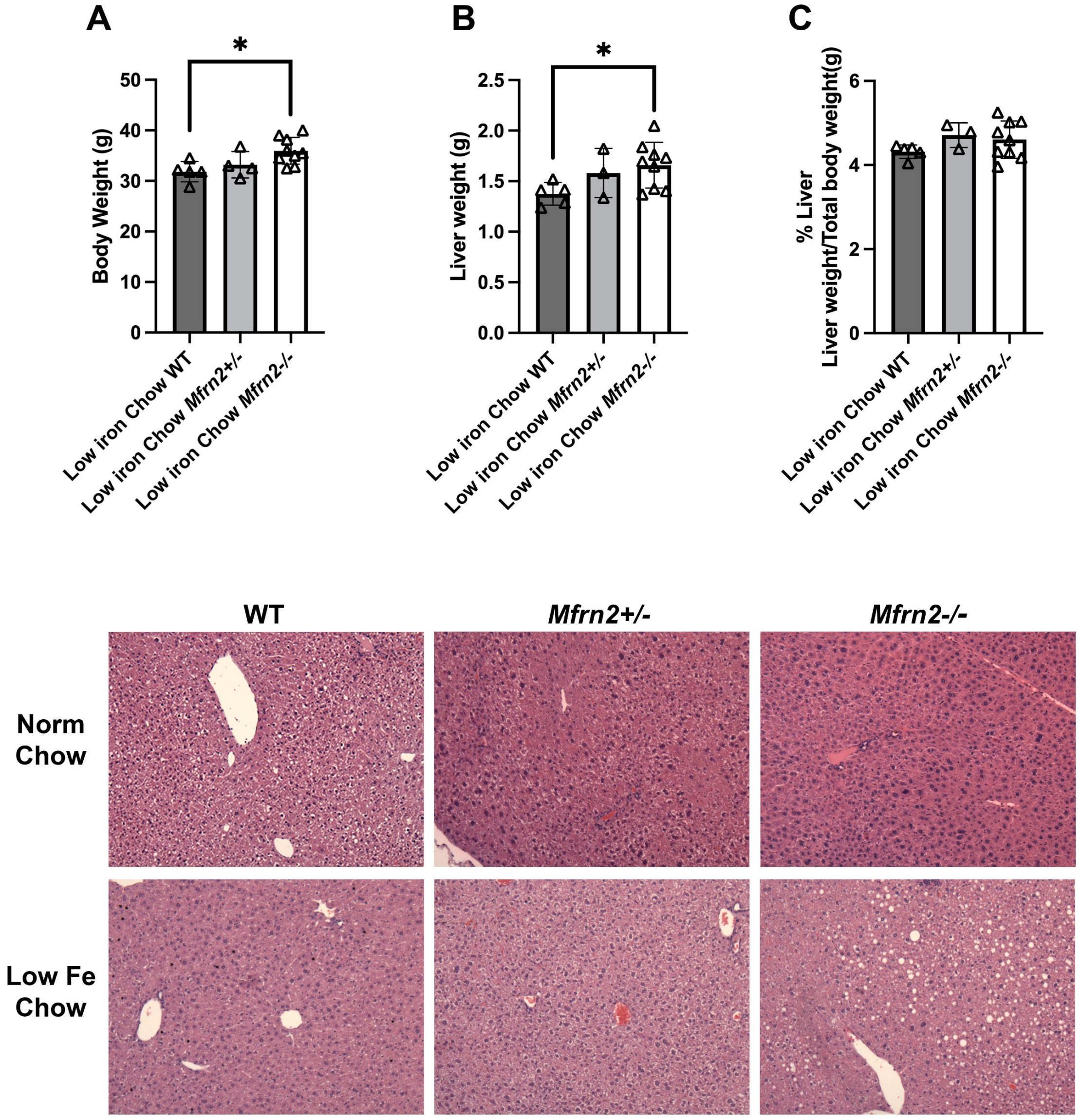

Supplemental Figure S1 The low iron diet reveals $Mfrn2^{-/-}$ male mice also show MASLD.

Six to eight week old male *Mfrn2*$^{+/+}$, *Mfrn2*$^{+/-}$, and *Mfrn2*$^{-/-}$ mice were maintained on a normal chow (350 mg/kg iron) or a low iron chow (4-8 mg/kg iron) for 10 weeks (n=3-9 mice/genotype/diet). Low iron diet (a) body weight, (b) liver weight and (c) %Liver ratio (liver/body weight. (d) H&E staining on 5 μ liver sections was performed from animals on each diet and images captured using a 10X objective. Representative images are shown for each genotype on each diet. A representative experiment is shown. For all graphs, statistics were performed as described in Materials and Methods with $p \leq 0.05$ considered significant.

Sup Fig 2

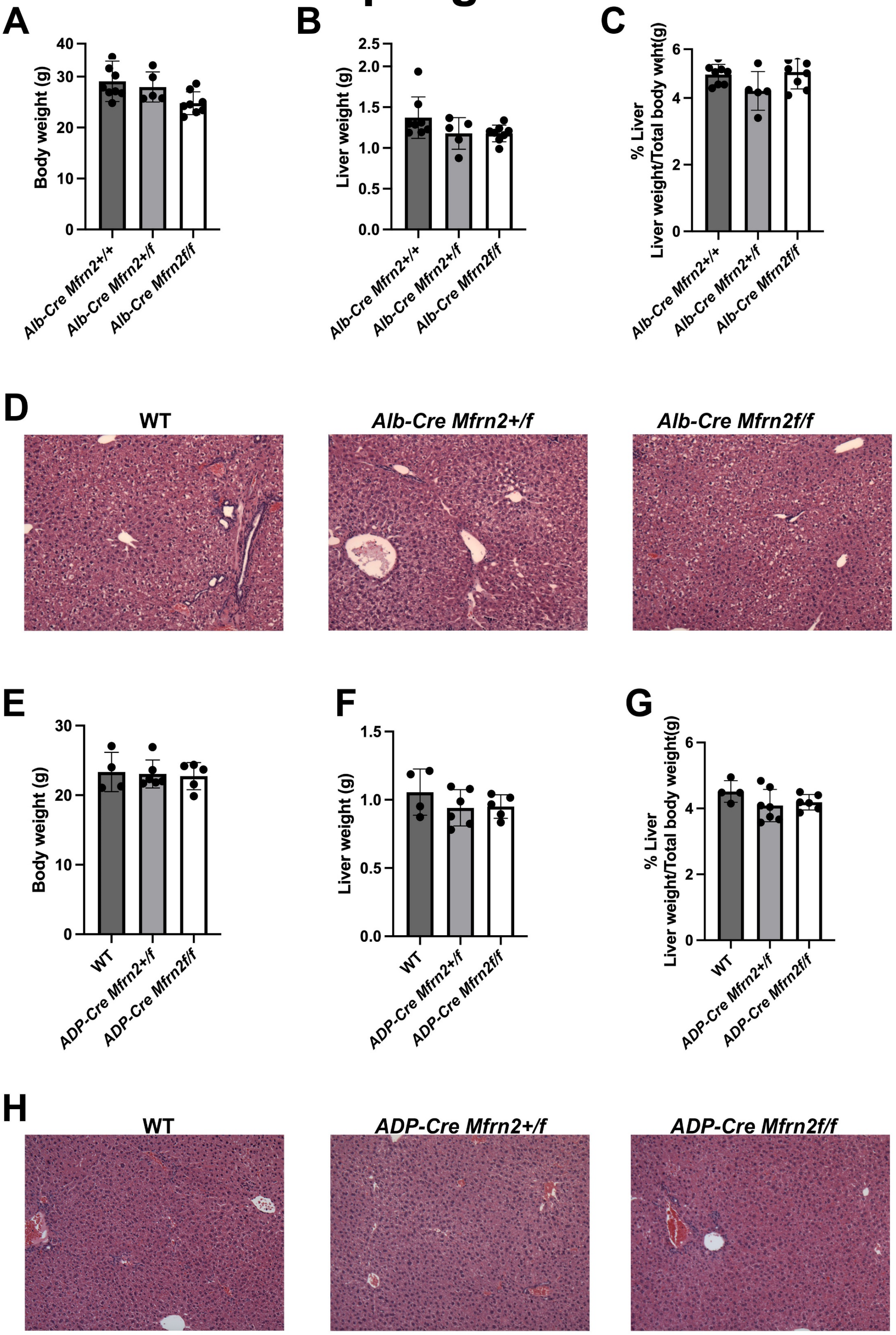

A
B
C
Body weight (g)
Liver weight (g)
% Liver
Liver weight/Total body weight(g)
Alb-Cre Mfrn2+/+
Alb-Cre Mfrn2+/f
Alb-Cre Mfrn2f/f
D
WT
Alb-Cre Mfrn2+/f
Alb-Cre Mfrn2f/f
E
F
G
Body weight (g)
Liver weight (g)
% Liver
Liver weight/Total body weight(g)
WT
ADP-Cre Mfrn2+/f
ADP-Cre Mfrn2f/f
H
WT
ADP-Cre Mfrn2+/f
ADP-Cre Mfrn2f/f

Supplemental Figure S2 Tissue specific loss of Mfrn2 in hepatocytes or adipocytes does not result in MASLD

Alb-Cre SPF and *Alb-Cre Mfrn2*$^{-/-}$ female mice were fed a normal chow diet as in figure 4 and a) Body weight, (b) Liver weight, (c) %Liver/Body weight and (d) liver histology examined. (n=5-8). *ADP-Cre* SPF and *ADP-Cre Mfrn2*$^{-/-}$ mice were treated as in a and (e) Body weight, (f) Liver weight, (g) %Liver/Body weight and (h) liver histology examined. (n=4-6). Representative images are shown for each genotype on the normal chow diet. A representative experiment is shown. For all graphs, statistics were performed as described in Materials and Methods with $p \leq 0.05$ considered significant.

Sup Fig 3

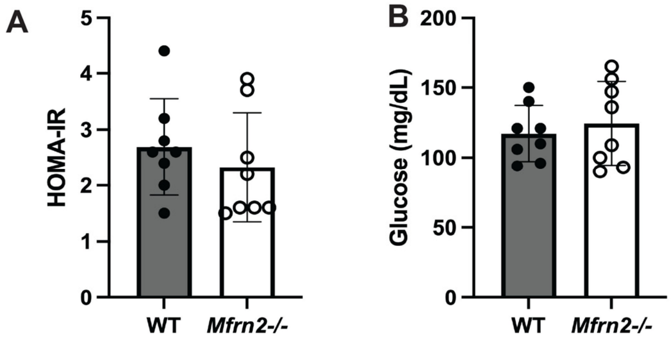

Supplemental Figure S3 Loss of Mfrn2 does not affect serum glucose levels nor HOMA-IR

HOMA-IR (A) and Serum glucose (B) from animals as in figure 4 were determined. A representative experiment is shown. N=2-3 separate experiments. For all graphs, statistics were performed as described in Materials and Methods with $p \leq 0.05$ considered significant.
